# Optimizing Intestinal Drug Delivery: A Comparative Study of Commercial Enteric Capsules and 3D-Printed Capsules with Customizable Release Profiles for Enhanced Precision Medicine

**DOI:** 10.3390/ma19030532

**Published:** 2026-01-29

**Authors:** Devansh Sharma, Shantanu G. Gaurkhede, Jia Deng, Anthony J. Di Pasqua

**Affiliations:** 1 Department of Pharmaceutical Sciences, Massachusetts College of Pharmacy and Health Sciences, Boston, MA 02115, USA; 2 School of Systems Science and Industrial Engineering, The Thomas J. Watson College of Engineering and Applied Science, Binghamton University, Binghamton, NY 13902, USA; 3 Department of Computer Science and Engineering Technology, Valdosta State University, Valdosta, GA 31698, USA

**Keywords:** 3D-printed capsules, 3D printing in drug delivery, controlled drug release, fused filament fabrication (FFF), enteric coating alternatives, hydroxypropyl methylcellulose acetate succinate (HPMC-AS), precision medicine, biodegradable polymers in pharmaceutics

## Abstract

**Highlights:**

**What are the main findings?**

- 3D-printed capsules provided zero drug release in gastric pH for 2 h.
- Controlled, sustained acetaminophen release achieved in the intestinal phase up to 5 h.
- Polymer ratio (HPMC-AS:PEG) directly tuned the release rate and duration.
- Printed capsules met the pharmacopeial weight and uniformity criteria.
- Korsmeyer–Peppas model indicated diffusion + erosion release mechanism.

**What are the implications of the main findings?**

- Demonstrated feasibility of patient-specific, tunable oral dosage forms via 3D printing.
- Enabled personalized, sustained-release therapy without traditional enteric coating.
- Simplified capsule fabrication into a one-step process integrating protection and control.
- Supports the future of on-demand drug manufacturing for precision medicine.
- Potential to improve adherence and reduce side effects in chronic therapy.

**Abstract:**

Conventional gelatin capsules deliver a rapid drug release in the stomach, which is suboptimal for therapies requiring controlled and delayed release, emphasizing the need for customizable drug delivery systems for precision medicine. This study’s objective was to optimize 3D-printed capsule shells formulated with pH-responsive polymer blends—hydroxypropyl methylcellulose acetate succinate (HPMC-AS), PEG-4000, and PVA—to achieve controlled and sustained drug release, comparing profiles against a commercial enteric capsule. Capsule shells were produced via fused filament fabrication (FFF) at two ratios (80:15:5 and 70:20:10), filled with acetaminophen (250 mg), and tested using a two-stage dissolution method (simulated gastric fluid (SGF) for 2 h followed by simulated intestinal fluid (SIF) for 4–5 h). Results showed negligible drug release in SGF (≤5%) for both printed and commercial capsules. However, in SIF, the commercial capsule released its payload rapidly (>80% within 15 min), while the 3D-printed capsules achieved a prolonged, gradual release. The higher HPMC-AS content significantly extended the release duration. All capsules met the pharmacopeial weight uniformity criteria. In conclusion, the 3D-printed shells provided a controllable, sustained drug release profile, underscoring 3D printing’s potential to create tunable, patient-specific dosage forms.

## 1. Introduction

Modified-release dosage forms are a drug delivery approach designed to release the drug over time or at a specific location within the body. This method aims to achieve maximal pharmacological effects while minimizing unwanted side effects, thereby improving safety and patient compliance in targeted therapy [1,2]. Oral capsule drug delivery is one of the most common pharmaceutical modalities due to its ability to accommodate various forms of drugs, including solid, semisolid, and liquid forms. However, conventional capsule shells (typically made of gelatin) have inherent limitations for controlled drug release [3]. Standard gelatin capsules typically disintegrate rapidly in the stomach, leading to rapid drug release that may not be ideal for drugs requiring sustained exposure [3,4,5].

To address this, conventional manufacturing utilizes the enteric coating of tablets and capsules for modified-release drug delivery systems. These capsules are designed to prevent drug release in the acidic environment of the stomach, ensuring that the drug is released in the more alkaline pH of the small intestine. This is particularly beneficial for acid-labile drugs that are sensitive to degradation in the stomach. However, the process of enteric coating utilizes a cellulose acetate phthalate (CAP) polymer, which must be treated with organic solvents to be used in the dip coating of capsules. The use of organic solvents presents risks as they are easily flammable, toxic, and harmful to the environment, and may leave residuals in the final product. Additionally, gelatin is an animal-derived material with dietary and stability concerns (e.g., restrictions for certain patient diets and a tendency for cross-linking), which has spurred interest in non-gelatin alternatives [6].

Moreover, poorly tunable “one-size-fits-all” release profiles can result in suboptimal therapy, particularly for drugs with narrow therapeutic windows or for patients who would benefit from tailored dosing schedules [7,8,9]. Indeed, individualized drug delivery has been shown to improve efficacy and safety, but current mass manufacturing methods are not well-suited to producing patient-specific dosage forms [4,10]. To address these issues, research efforts are increasingly focused on developing capsule technologies that can target release to specific gastrointestinal (GI) locations and sustain drug release over desired timeframes [8,11]

Additive manufacturing, or 3D printing, has emerged as a revolutionary tool in pharmaceutical sciences, enabling on-demand fabrication of dosage forms with complex geometries and tunable properties [3,10,12,13,14,15]. Unlike traditional mass-production methods, 3D printing enables the customization of dosage forms in terms of drug content, shape, and release characteristics for individual patients [12,16,17,18]. Notably in 2015, the U.S. FDA approved the first 3D-printed drug (Spritam^®^ levetiracetam), demonstrating the ability of the 3D printing process to produce a porous, rapidly disintegrating tablet that is unattainable by conventional techniques [7,19]. Spritam’s high drug-loaded, highly porous structure enables it to disperse in seconds and reach therapeutic levels in minutes, benefiting epilepsy patients who need a fast-acting, easy-to-swallow medication [7,19,20,21]. This milestone underscored the potential of 3D printing to transform medication design, prompting extensive research into 3D-printed tablets, multi-drug polypills, and capsules for customized therapy [12,14,16,17]. Recent studies highlight that 3D-printed medicines can incorporate multiple drugs and achieve precise dosing, and that one can modulate release profiles by varying the formulation or structural parameters [10,12,22,23]. For example, adjusting the infill density of 3D-printed tablets has been shown to strongly influence drug release kinetics: low-density (20% infill) constructs release the drug rapidly, whereas high-density (80% infill) constructs prolong release due to reduced porosity [24].

Among the 3D printing techniques, fused filament fabrication (FFF) is one of the most widely adopted methods for pharmaceuticals due to its ability to create customized oral dosage forms using filament-based polymers [13,14,25]. FFF involves melting a thermoplastic material filament and depositing it layer by layer, which is particularly suitable for fabricating capsule shells that need mechanical integrity and controlled disintegration [25]. Prior studies have explored FFF-printed capsules for various applications. For instance, multi-compartment “polypill” capsules containing up to five different drugs were fabricated in separate sections, achieving complex release patterns in a single device [17,26]. Capsules with pH-responsive properties were developed via FFF using enteric polymers, demonstrating the feasibility of protecting a payload in stomach-like conditions and releasing it at higher pH [27]. In pediatric medicine, 3D printing has been utilized to enhance dosing and compliance. For instance, candy-like chewable tablets (shaped as popular Starmix^®^ gummy candies) that masked the bitter taste of a drug have been printed, making it more palatable for children [28]. These pediatric formulations achieved excellent taste-masking and enabled personalized dosing in child-friendly formats [28]. Similarly, patient-specific enteric capsules for the non-steroidal anti-inflammatory drug (NSAID) ketoprofen have been reported, demonstrating that adjusting shell polymer composition and thickness allows for tuning the lag time and release rate to meet individual needs [29]. In our own previous work, we found that 3D-printed capsules made of PVA and HPMC (a hydrophilic polymer) released acetaminophen significantly more slowly than standard gelatin capsules [3]. Notably, increasing the HPMC content from 5% to 25% in the printed shell composition progressively delayed the drug’s release, an effect that was associated with smoother plasma concentration profiles and potentially reduced side effects [3,30]. This prior study demonstrated that polymer compositions can be leveraged to modulate dissolution rates in 3D-printed capsules, pointing toward the value of tailoring materials for desired release characteristics [3,30]. However, a systematic optimization of polymer blends for achieving both gastric protection and sustained intestinal release, in direct comparison with commercial enteric capsules, remains an open research question.

This study addresses that gap by optimizing 3D-printed capsule shells formulated with blends of an enteric polymer, HPMC-AS, and hydrophilic additives, PEG and PVA, to tailor drug release profiles [30]. HPMC-AS is a pH-sensitive polymer widely used in enteric coatings, dissolving at pH > 5.5 [29]. By incorporating HPMC-AS into the capsule shell matrix, we aimed to prevent drug release in the stomach and allow the shell to gradually dissolve or erode in the intestine with controllable release rates [11,15]. PEG was included as a pore-forming plasticizer to adjust shell hydrophilicity, while a small amount of PVA ensured the printability and mechanical strength of the filament [31]. Two different HPMC-AS/PEG/PVA ratios were investigated to evaluate how increasing the fraction of hydrophilic polymer (PEG) versus enteric polymer (HPMC-AS) influences dissolution behavior [32]. The performance of these custom 3D-printed capsules was benchmarked against a commercial enteric-coated gelatin capsule, “XPRS Nutra Size 1”, which was designed to remain intact in stomach acid and release rapidly in the intestine.

We hypothesize that 3D-printed capsules can be engineered to achieve a more gradual, sustained release in the intestinal phase than standard enteric-coated capsules by virtue of a bulk-eroding polymer matrix as opposed to a thin enteric film coating [8,11]. Such capability would be highly valuable for precision medicine, allowing clinicians or pharmacists to print patient-specific capsules that deliver drugs at the desired rate and target site [16,17,18]. In the following sections, we describe the design and fabrication of the capsules, the characterization of their dissolution profiles in vitro, and a comparative analysis highlighting the advantages of the optimized 3D-printed shells. The broader implications for personalized drug delivery and pharmaceutical manufacturing are also discussed.

## 2. Materials and Methods

### 2.1. Materials and Filament Preparation

Pharmaceutical-grade polymers and excipients were selected to produce capsule shells with enteric and delayed-release functionality. Hydroxypropyl methylcellulose acetate succinate (HPMC-AS, LF grade), a pH-dependent polymer that remains intact in gastric pH but dissolves above pH 5.5, was obtained from Sigma-Aldrich (St. Louis, MO, USA). Poly(ethylene glycol)-4000 (PEG-4000) was also sourced from Sigma-Aldrich. Poly(vinyl alcohol) (PVA) filament (3.00 mm diameter, pharmaceutical grade) was purchased from Ultimaker (Geldermalsen, The Netherlands). All materials were used as received and were chosen based on prior evidence of their effectiveness in modulating drug release profiles and suitability for extrusion-based 3D printing. The active pharmaceutical ingredient (API) used in this study was acetaminophen (model drug for analgesic/antipyretic), which was derived from commercially available Timely^®^ Extra Strength caplets (Time-Cap Labs, New York, NY, USA) (500 mg each), and commercial capsules shells were sourced from XPRS Nutra Enteric empty gelatin capsules (XPRS Nutra, Jordan, UT, USA, Lot Number: X0037YPE9T, Expiry Date: 31 December 2026). The caplets were pulverized using a mortar and pestle, and the resulting powder was sieved through 20-mesh and 40-mesh screens to ensure a uniform particle size for capsule filling. To prepare printable filaments, two custom polymer blend formulations were developed as shown in Table 1:

The compositions were chosen to examine the effect of increasing PEG (from 15% to 20%) and PVA (5% to 10%) while reducing HPMC-AS, hypothesizing that Blend B’s higher hydrophilicity would accelerate drug release compared to Blend A. These two blends were selected based on the processing and functional limits established in our previous research work [15]. Our prior study identified that an HPMC-AS content exceeding 80% (as in Blend A) induces extreme filament brittleness, which prevents successful feeding and 3D printing. On the other hand, decreasing the HPMC-AS content below 70% (as in Blend B) was found to compromise the enteric barrier, leading to premature drug release during the gastric acid phase. Within this 70–80% window, the plasticizer ratios were strategically optimized for successful filament extrusion and capsule fabrication. For example, increasing PEG beyond 20% resulted in overly soft filaments that deformed during extrusion, while lowering it below 15% at high polymer loads led to nozzle clogging. Consequently, Blends A and B represent the feasible upper and lower boundaries of the formulation window for these polymer formulations, providing a comprehensive characterization of the achievable release spectrum [15]. Each blend (total ~10 g) was compounded by thoroughly mixing the powdered constituents and then feeding them into a single-screw hot-melt extruder to form filaments. Filament extrusion was carried out at ~170–180 °C (a temperature sufficient to soften HPMC-AS and PVA) using a custom lab-scale extruder (EX2, Filabot, Barre, VT, USA). The extruded filament was 1.75 mm in diameter, matching the FFF printer (Prusa i3 mk3s, Prusa Research, Prague, Czech Republic), and was cooled and spooled for subsequent printing. The mechanical properties of the filaments (flexibility, strength) were qualitatively checked to ensure smooth feeding into the 3D printer. All filaments were stored in airtight bags with desiccant until use to prevent moisture uptake by the hygroscopic polymers.

### 2.2. Capsule Design and 3D Printing

**Figure 1:**
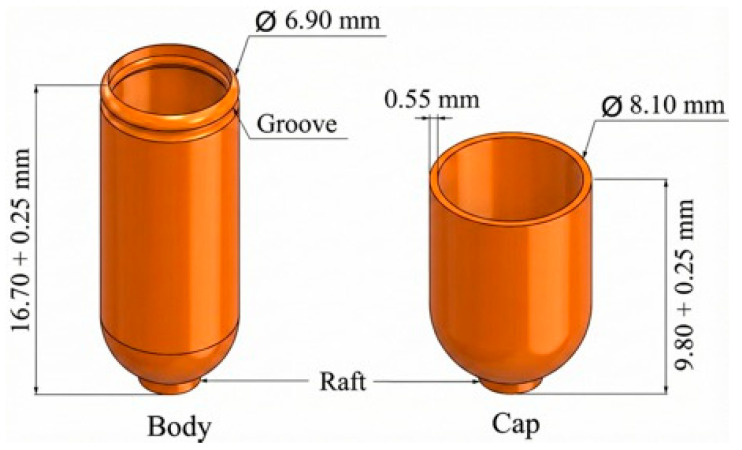
Designed size 0 capsule.

Capsule shells were designed in two parts (cap and body), analogous to standard two-piece capsules. A computer-aided design (CAD) model of a size 0 capsule (approximately 19.4 mm length, 6.9 mm diameter) was created, and the shell thickness was set at 0.6 mm to ensure robustness and accommodate the FFF printing resolution. Figure 1 shows the designed capsule and its dimensions.

The 3D models were exported as STL files and sliced using Prusa Slicer software (software version 2.6). Printing was performed on an FFF printer equipped with a 0.4 mm nozzle. The printer was calibrated to ensure dimensional accuracy and consistent extrusion. Print settings were optimized for pharmaceutical printing and preliminary trials and are shown in Table 2. A brim was used to improve bed adhesion. Each capsule half was printed in an upright orientation (open end facing up) to achieve a uniform wall thickness.

The printed capsule halves were allowed to cool and then examined. The actual printed capsules are shown in Table 2. All capsules showed smooth surfaces and intact structures, with no visible warping or defects. Ten full capsules of each 3D-printed batch (Blend A and Blend B) were produced for testing. Additionally, commercial enteric capsules (XPRS Nutra, Size 1, white hard gelatin with enteric coating) were used as the comparator. These commercial shells are composed of gelatin with a proprietary enteric polymer coating (designed to resist dissolution at pH 1.2 and dissolve at pH ≥ 5.5).

### 2.3. Thermogravimetric Analysis (TGA)

Thermogravimetric analysis was performed on Blends A and B using TGA Q50 (TA Instruments, New Castle, DE, USA). The instrument calibration was performed at 25 °C with a 20 °C/min ramp rate until 500 °C. Nitrogen was used as the purge gas with a flow rate of 50 mL/min. Finally, TA Instrument Universal V4.5A software was used to analyze the thermal degradation of polymer blends at HME and FFF operating temperatures.

### 2.4. Capsule Filling and Weight Variation

Prior to filling, empty printed capsule shells were pre-screened for consistency in weight. The average weight of an empty 3D-printed capsule (cap and body) was ~373.2 mg for Blend A and ~361.2 mg for Blend B, reflecting the slightly greater material usage in Blend A (due to higher density and HPMC-AS content). The enteric-coated gelatin capsules (empty) weighed ~107.68 mg each (with coating). Each capsule was manually filled with acetaminophen powder to achieve a target fill weight of 250 mg API using a spatula and the tapping method. Care was taken to pack the powder uniformly. After filling and capping, the total weight of each capsule was measured on an analytical balance. All commercial capsules were within ±5% of the intended total weight of 385–390 mg (shell and powders), satisfying the USP weight uniformity requirements for capsules of this fill mass. The relative standard deviation (RSD) of filled capsule weight was <5% for both 3D-printed batches and for the commercial capsules, indicating good content uniformity. Capsules were stored in dry conditions at room temperature for at least 24 h prior to dissolution testing, allowing the powder to settle and ensuring consistent initial conditions.

### 2.5. Dissolution Testing Protocol

In vitro dissolution studies were conducted using a USP Type 1 basket apparatus (Distek 2500 Premiere Dissolution Bath System, Distek, NJ, USA) with six vessels, following standard guidelines for delayed-release capsules [33,34,35]. The study employed a two-stage dissolution test to simulate gastrointestinal transit: Stage I (acid phase) in simulated gastric fluid (SGF, pH 1.2) for 2 h, followed by Stage II (intestinal phase) in simulated intestinal fluid (SIF, pH 6.8) for up to an additional 4–5 h. This pH change method (acid → buffer) is recommended for evaluating enteric-coated formulations and was used to assess both the integrity of capsule shells in stomach-like conditions and the subsequent drug release profile in intestine-like conditions [33,34,35].

For the SGF, 900 mL of 0.1 N HCl (prepared by diluting concentrated HCl with distilled water and adding 2.0 g/L NaCl) was used in each vessel (37 ± 0.5 °C). Each capsule (*n* = 6 per formulation) was placed in a stainless-steel basket and lowered into the SGF medium, rotating at 100 rpm. After 2 h, the dissolution media were replaced with SIF by adding 900 mL of Potassium Phosphate Monobasic (KH_2_PO_4_) and sodium hydroxide (NaOH), 0.2 M solution, to raise the pH to 6.8. The rotation speed was maintained at 100 rpm throughout. Aliquots (5 mL) were withdrawn at predetermined time points: 15, 30, 60, 90, and 120 min during the SGF stage, and at 15 min (i.e., 135 min overall, shortly after pH switch), 30 min, 1 h, 2 h, 3 h, 4 h, and 5 h during the SIF stage. An early 15 min sample in SIF was included to capture the initial release right after the pH transition, which is critical for detecting any burst release. For each sample withdrawn, an equal volume of pre-warmed medium was added to the vessel to maintain a constant volume.

All samples were immediately filtered through 0.45 µm PTFE syringe filters (VWR International, Radnor, PA, USA) to remove any undissolved particles or shell fragments. Filtrate (2 mL) was analyzed for acetaminophen concentration using a Cary 50 UV–Vis spectrophotometer (Agilent Technologies, Santa Clara, CA, USA) at λ = 243 nm (the absorption maximum for acetaminophen in these media). The spectrophotometer was blanked with SGF or SIF as appropriate, and a calibration curve was prepared using standard acetaminophen solutions in each medium (concentration range 1–50 µg/mL, R^2^ > 0.999). The percent cumulative release of acetaminophen was calculated at each time point by normalizing the measured concentration to the commercial capsules’ drug content (250 mg). No interference from capsule polymers was observed at 243 nm. The dissolution run included all formulations tested in parallel (printed Blend A (*n* = 3), printed Blend B (*n* = 3), and commercial capsule (*n* = 6)).

### 2.6. Data Analysis and Modeling

The dissolution efficiency (DE) up to 6 h was calculated as the area under the dissolution curve (AUC) expressed as a percentage of the area corresponding to 100% release over the same duration. To quantitatively compare the 3D-printed and commercial capsule profiles, the similarity factor f_2_ was computed for each pair of profiles (using averaged data) according to FDA guidelines. An f_2_ value between 50 and 100 indicates a similarity in release profiles, whereas a lower value indicates dissimilarity. Because the three groups had unequal sample sizes (*n* = 6, 3, 3) and exhibited heteroscedastic variances, differences in percent drug release at shared time points were evaluated using Welch’s ANOVA, followed by Games–Howell post hoc tests to identify pairwise differences. This approach does not assume equal variances and is appropriate for unbalanced designs, ensuring that reported *p*-values reflect the true variability of each group. A *p*-value < 0.05 was considered statistically significant.

Additionally, the dissolution data were fitted to common kinetic models (zero-order, first-order, Higuchi, Korsmeyer–Peppas) to elucidate the release mechanisms. The best-fit model was selected based on the highest R^2^ and lowest Akaike Information Criterion (AIC). Model fitting and statistical analyses were performed using Microsoft Excel and GraphPad Prism (Software version 10.5.0), and data are reported as mean ± standard deviation unless otherwise specified. Representative dissolution curves with error bars (mean ± SD) were plotted for each formulation.

## 3. Results

### 3.1. Dissolution Profiles in SGF and SIF

**Figure 2:**
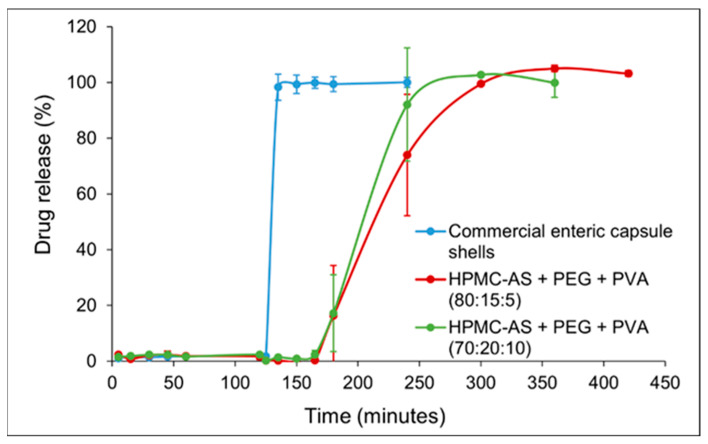
Dissolution profiles of acetaminophen from 3D-printed capsules (Blend A: 80:15:5 HPMC-AS/PEG/PVA; Blend B: 70:20:10 HPMC-AS/PEG/PVA) compared to a commercial enteric-coated gelatin capsule.

Capsules were subjected to 2 h in simulated gastric fluid (SGF, pH 1.2) followed by 2–5 h in simulated intestinal fluid (SIF, pH 6.8), Figure 2. The plotted values represent the mean percentage of cumulative drug released (*n* = 6 for the commercial capsule and *n* = 3 for each 3D-printed formulation), with error bars showing the standard deviation. In SGF (0–120 min): The commercial enteric capsule (blue curve) exhibited negligible drug release (0–2%) over the 2 h acid stage, confirming the effectiveness of its enteric coating in preventing acetaminophen release at low pH. Importantly, both 3D-printed capsules closely mimicked this gastric protection, releasing only 2.37 ± 1.13% (Blend A) and 2.31 ± 0.94% (Blend B) by 120 min with no statistically significant difference from the commercial capsule (Welch-type comparison, *p* > 0.05). No capsule rupture or disintegration was visually observed for any capsule in SGF, indicating that the printed HPMC-AS/PVA matrices, like the enteric-coated gelatin shells, remained intact in the acidic environment and thereby fulfilled the prerequisite “no-release-in-stomach” criterion necessary for targeted intestinal delivery.

In SIF (after 120 min): Upon transition to pH 6.8 at t = 120 min, the dissolution profiles diverged markedly between the commercial capsule and the 3D-printed capsules. The enteric-coated gelatin capsule released its payload rapidly once in the intestinal medium. As shown in Figure 2 (blue curve), the commercial capsule released ~98% of acetaminophen within the first 15 min in SIF. The release plateaued with ~95% of the drug dissolved by 15 min, indicating a typical delayed immediate-release behavior—a lag in acid followed by a prompt release in buffer. In contrast, the 3D-printed capsules achieved a slower, sustained release in the SIF phase. Blend A (80% HPMC-AS, 15% PEG; red curve) released ~17% by 1 h into SIF (t = 180 min) and ~73% by 2 h in SIF (t = 240 min). Thereafter, drug release continued gradually: ~99% by 300 min, ultimately reaching ~100% by 420 min (5 h in SIF). Blend B (70% HPMC-AS, 20% PEG; green curve) released faster than Blend A, consistent with its higher PEG content. Within 2 h of SIF exposure (t = 240 min), Blend B had released ~92% (vs. ~73% for Blend A). Blend B reached ~100% cumulative release by 300 min total (i.e., 3 h in SIF), slightly earlier than Blend A. This demonstrates that the release lag and rate can be tuned simply by adjusting the polymer composition, paving the way for patient-specific dosage forms designed to target drug release to specific intestinal segments or therapeutic windows. Despite the faster rate relative to Blend A, Blend B still provided a more extended release than the commercial capsule, which had essentially completed release by 15 min in SIF.

These results demonstrate that all capsule types successfully prevented drug release in the gastric phase (no premature acetaminophen leakage in SGF) and that the 3D-printed capsules can modulate the post-pH-switch release kinetics through formulation differences. The sustained-release effect was especially pronounced for the high-HPMC Blend A, whereas Blend B showed a moderately faster release but was still not as abrupt as the commercial product. The dissolution curves of both 3D-printed formulations were smooth and sigmoidal, lacking the sharp upswing observed with the enteric gelatin capsule. This suggests a controlled diffusion/erosion mechanism at play rather than an immediate disintegration.

Statistical analysis further substantiated these findings. Welch’s ANOVA revealed a highly significant group effect at 135 min (F ≈ 520, *p* < 0.001), confirming that the release behavior differed among formulations. Games–Howell post hoc comparisons demonstrated that the commercial capsule released significantly more drug than both Blend A (adjusted *p* < 0.000000009) and Blend B (adjusted *p* < 0.00000004) at this early SIF time point. At later time points, Blend B showed a statistically significantly faster release compared to Blend A (e.g., at 240 min, *p* < 0.05), reinforcing the conclusion that composition can be used to control release rate. The dissolution efficiency (0–6 h) was highest for the commercial capsule (~88.5%), intermediate for Blend B (~77.3%), and lowest for Blend A (~65.1%), reflecting the greater extent of sustained release in the latter. The mean dissolution time (MDT) was significantly prolonged for Blend A vs. the commercial (*p* < 0.01), confirming its extended-release character. The similarity factor f_2_ calculations yielded f_2_ ≈ 12.8 when comparing the commercial capsule to Blend A, and f_2_ ≈ 13.3 for commercial vs. Blend B, indicating that the 3D-printed profiles are dissimilar to the commercial profile (f_2_ < 50). In contrast, the two 3D-printed profiles had an f_2_ of ≈61.2 when compared to each other, confirming that while Blend A and B differed in rate, their overall release pattern (delayed and sustained) was more similar to each other than to the rapid-release commercial capsule. These statistically robust findings highlight that we were able to achieve 3D printing not only for reliable gastric protection but also programmable, composition-dependent intestinal release—a key step toward the rational design of patient and disease-specific oral drug delivery systems aimed at maximizing therapeutic efficacy and minimizing adverse effects.

### 3.2. Weight Variation and Content Uniformity

All capsules, both 3D-printed and commercial, exhibited acceptable weight uniformity. The 3D-printed Blend A capsules (filled) weighed 652.9 ± 27.4 mg on average, and the Blend B capsules weighed 640.4 ± 31.2 mg compared to commercial enteric capsules at 357.8 ± 1.4 mg (mean ± SD, *n* = 3, 3, and 6, respectively). The higher weight of the printed capsules is attributed to the denser polymer shell matrix. The powder fill weight (target ~250 mg) was consistent across all capsule types, with an RSD of 4.2% for Blend A, 4.9% for Blend B, and 0.4% for the commercial capsules. These values are well within the USP uniformity of the dosage units’ criteria, which typically allows for ±5% deviation for capsules of this fill size. There were no signs of weight loss or leakage after the dissolution test, indicating that the capsule shells remained intact through the acid stage and only dissolved/eroded in the intestinal stage as intended. The uniform filling of the 3D-printed capsules demonstrates that they can be handled and loaded with drug powders in a manner similar to that of standard capsules without significant content variability.

### 3.3. Mechanistic Release Analysis

The dissolution data from the 3D-printed capsules suggest a combination of diffusion and polymer erosion mechanisms controlling drug release. To further investigate this, the release profiles were fitted to various kinetic models, including zero-order, first-order, second-order, Higuchi, and Korsmeyer–Peppas models. The goodness-of-fit for each model is summarized in Table 3. As shown in the table, neither pure zero-order nor simple first-order kinetics described the data well for the 3D-printed capsules. The Higuchi model, which describes diffusion-controlled release, provided a better fit, indicating a significant contribution of diffusion. However, the Korsmeyer–Peppas model provided the best fit for both Blend A and Blend B (R^2^ > 0.99), with release exponent values (n ≈ 0.66) indicating anomalous (non-Fickian) transport. This confirms that a coupling of drug diffusion governs acetaminophen release through the gradually hydrating polymer matrix and slow dissolution/erosion of the HPMC-AS polymer network [3]. In contrast, the commercial capsule’s release was best described by second-order kinetics, although a first-order model also provided a reasonably good fit. This is likely due to the rapid disintegration of the gelatin shell after the enteric coating dissolved, leading to immediate exposure of the drug and fast dissolution. In the SGF stage, the HPMC-AS polymer does not ionize or swell significantly (being insoluble at pH 1.2), thus effectively trapping the drug. Once in SIF, the HPMC-AS begins to ionize and dissolve, especially at the surface of the capsule, while PEG dissolves quickly, creating aqueous channels. Blend A, with a higher HPMC-AS content, maintains its matrix integrity longer, consistent with a sustained, erosion-controlled release. Blend B, containing more PEG, likely develops pores faster, facilitating quicker diffusion of acetaminophen out of the matrix (hence the slightly higher initial slope in SIF and higher k value). The commercial capsule, on the other hand, released acetaminophen largely through the disintegration of the gelatin shell once the enteric coating dissolved at ~pH 6.8. The disintegration of the shell resulted in an immediate exposure of the entire powder bed to the fluid, causing a near first-order dissolution of the drug (approaching completion within ~15 min in SIF).

It is noteworthy that a significant lag time was observed for the 3D-printed capsules upon transitioning to pH 6.8. While drug release commenced in SIF, it did so at a highly controlled rate, demonstrating the effectiveness of the polymer matrix in delaying release. This indicates that the capsule shells began to permit fluid ingress or polymer relaxation after the pH change, but without cracking or bursting. The data point at t = 135 min (15 min into SIF) showed minimal release for the printed capsules (generally below 2%), confirming the delayed onset of release. The enteric-coated gelatin capsule, in contrast, had already released the vast majority of its contents prior to entering SIF due to the dissolution of the enteric coating in the acidic phase. By 150 min (30 min into SIF), the difference was stark: the commercial capsule was nearly completely released, whereas the 3D-printed capsules still exhibited very limited drug release, highlighting the sustained-release properties of the 3D-printed formulations.

The ability to modulate release kinetics through formulation is clearly demonstrated by comparing the Batch 1 (Blend A) and Batch 2 (Blend B) 3D-printed capsules. Both provided the desired biphasic behavior—negligible release in acid, followed by substantial release in neutral pH—fulfilling the targeted release objective. However, by simply altering the HPMC-AS:PEG ratio, we achieved either a more prolonged release (with 80% HPMC-AS) or a moderately faster-sustained release (with 70% HPMC-AS). This tunability is a key advantage of the 3D printing approach. Traditional enteric-coated capsules have a limited ability to adjust the release rate: once the coating dissolves, the underlying capsule or tablet often releases the drug rapidly. In contrast, the bulk polymer matrix of a printed capsule can act as a release-modifying platform even after the initial enteric function is served. Essentially, the 3D-printed shell itself doubles as both the protection layer (in stomach) and the control-releasing matrix (in intestine).

During the dissolution tests, the physical integrity of the capsules was periodically observed. In SGF, all capsule types remained intact; the gelatin capsule’s enteric coating prevented it from dissolving, and the printed HPMC-AS capsules did not swell or deform noticeably in the acidic medium. Immediately after switching to SIF, a slight opacity change was seen in the HPMC-AS capsules, indicating the beginning of polymer hydration. The gelatin capsule’s enteric coat was seen to dissolve off within ~5 min in SIF, after which the gelatin vessels turned translucent and quickly opened. For the 3D-printed capsules, partial shell integrity persisted for a longer duration: fragments of the shell were present even 2–3 h into SIF for Blend A, whereas Blend B’s shell became fragmentary by ~1–2 h as it dissolved faster. These qualitative observations support the quantitative data on release rates. Importantly, no large undissolved chunks of polymer remained by the end of 5–6 h for either blend; the HPMC-AS/PVA material eventually dissolved or eroded fully, ensuring complete drug availability.

### 3.4. Thermogravimetric Analysis

**Figure 3:**
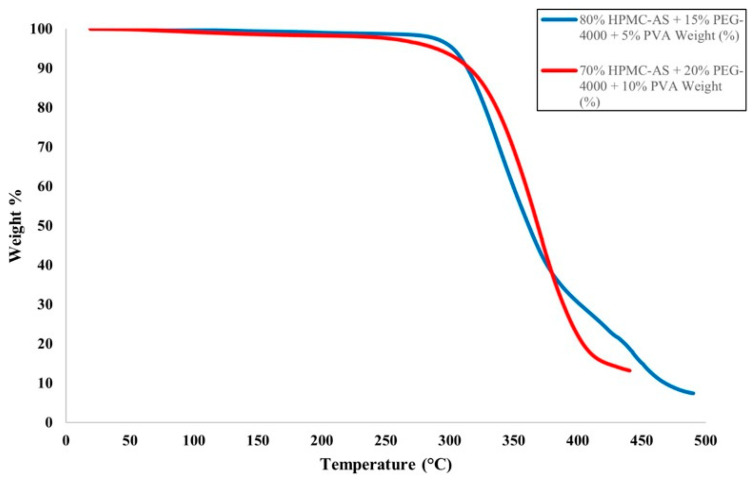
TGA spectra for the tested filament materials (Blend A: 80:15:5 HPMC-AS/PEG/PVA; Blend B: 70:20:10 HPMC-AS/PEG/PVA).

The HME and FFF processes involve high temperatures, which could lead to the degradation of the polymer materials used for the filament or the fabricated part. To prevent this, TGA was conducted on polymer blend materials, ensuring blends were processed below their degradation temperatures. The TGA spectra for the tested filament materials are presented in Figure 3. The filament prepared with 80% HPMC-AS, 15% PEG-4000, and 5% PVA (Blend A) showed less than 3% decomposition around 308 °C, exhibiting significant mass loss at higher temperatures. The filament prepared with 70% HPMC-AS, 20% PEG-4000, and 10% PVA (Blend B) exhibited less than 3% mass loss at 270 °C with significant mass loss over 278 °C. Both polymer blends showed primary mass loss ~270–310 °C, which is significantly higher than their processing temperatures in HME and FFF. Thus, none of the filaments underwent thermal degradation during the HME and FFF processes.

## 4. Discussion

Our findings demonstrate that 3D-printed capsule shells can be rationally engineered to achieve targeted and sustained drug release, addressing limitations of conventional capsule technologies. The optimized HPMC-AS/PEG/PVA capsules effectively withstood the gastric conditions and then released acetaminophen in a controlled, extended manner in the intestinal environment. In contrast, the benchmark enteric-coated gelatin capsules, while successfully delaying release in acid, delivered the drug in a rapid bolus upon reaching higher pH—a design suitable for enteric protection but not for sustained delivery. This direct comparison highlights a key advantage of 3D-printed oral dosage forms: by integrating functional polymers into the capsule’s bulk, one can obtain composite release profiles (e.g., delay + sustain) that are difficult to attain with standard coating approaches [4,36].

The concept of using polymer matrices for controlled release is well-established in tablet formulations, but its extension to capsule shells via 3D printing is relatively novel. Gaurkhede et al. similarly 3D-printed capsules from PVA blended with HPMC, and reported that acetaminophen dissolution from those printed capsules was significantly slower than from traditional gelatin capsules [3]. In their study, increasing the HPMC content from 5% to 25% in the capsule composition progressively delayed drug release, in line with our observations using HPMC-AS [3]. This work builds on this concept by employing HPMC-AS (an enteric polymer) at a much higher fraction (70–80%) to fully prevent any acid release, while still leveraging the HPMC-derived matrix to sustain release in neutral pH. Our previous study confirmed that this 70–80% range is the only feasible window for this system. Formulations outside this range failed either due to brittleness (>80%) or lack of acid resistance (<70%) [15]. The excellent acid resistance of our printed shells (virtually 0% release in 0.1 N HCl) is comparable to or better than other 3D-printed enteric systems reported. For instance, Eleftheriadis et al. developed FFF-printed capsules with Eudragit L100-55 (another enteric polymer) and achieved minimal release at pH 1.2 and complete release at pH 6.8 within 2–3 h [27]. Our HPMC-AS capsules prolonged the release further, to ~5 h, by virtue of the thicker, high-polymer-load shell acting as a sustained-release matrix.

It is informative to compare the release profiles to those of printed tablets with similar polymers. A study by Thakkar et al. (2020) used HPMC-AS in 3D-printed tablets and found that higher infill (denser structure) resulted in slower drug release [24], analogous to how our higher polymer (denser matrix) formulation (Blend A) released the drug more slowly than the one with more soluble filler (Blend B). In both cases, the design parameter (infill or formulation) effectively controlled the tortuosity of the matrix through which the drug diffuses [11,37]. These parallels indicate that fundamental release principles (diffusion/erosion control by polymer content and geometry) are consistent across different 3D-printed dosage form types.

A unique aspect of capsules is that they allow for the separation of formulation components between the shell and the fill. In our approach, we kept the fill as pure drug powder (as in a typical capsule) and instead modified the shell composition to control release. An alternative strategy demonstrated by Smith et al. is a “print-and-fill” capsule, where a pre-designed capsule structure is printed and then manually filled [38]. Smith et al. (2018) printed a single-step capsule shell with a cylindrical geometry, achieving satisfactory performance in terms of shell integrity and drug containment [38]. However, their focus was on the feasibility of printing and mechanical qualification of the capsule, not on sustained release. Our study takes this further by optimizing the shell material for controlled drug release. By doing so, we effectively turn the capsule shell into a functional delivery system, not just a container.

Another comparable work is the recent study by Shaqour et al., who printed patient-specific enteric capsules for ketoprofen release [29]. They investigated capsules made from hydroxypropyl methylcellulose phthalate (HPMCP) blended with PEG versus pure PVA, and observed that polymer type and capsule wall thickness critically influenced the drug release timing. Specifically, thicker HPMCP/PEG capsules delayed release longer (due to slower erosion) while PVA capsules released faster. These findings resonate with our results: HPMC-AS (chemically similar to HPMCP) provides the pH-triggered delay and erosion-controlled release, while the inclusion of PEG modulates the release rate by altering matrix porosity. Our two formulations mimic a “thick, strong enteric matrix” vs. a “more porous, faster-dissolving matrix”, analogous to their comparison between different capsule builds. Thus, across different studies, a clear picture emerges: polymer selection and ratio in 3D-printed capsules can tune the lag time and release rate, offering a versatile platform for modified-release drugs [3,29,38].

It is worth noting that traditional enteric-coated products usually aim for complete drug release shortly after the pH trigger, as the goal is typically to dump the dose in the intestine while avoiding the stomach [4]. For example, Delayed-Release (DR) formulations of acid-labile drugs (like proton pump inhibitors) or NSAIDs often dissolve entirely within 30–60 min post gastric transit. In contrast, the patient-tailored capsules we propose could be advantageous when a drug needs both protection from stomach acid and a controlled absorption profile in the gut. Potential applications might include chronotherapy (timing drug release to match circadian rhythms), reducing peak-related side effects by smoothing plasma levels, or delivering drugs to a specific GI region (e.g., colon) over an extended period [39]. While our study used acetaminophen as a model (which normally is immediate-release), the principle can be translated to other drugs. For instance, imagine a scenario in which a once-daily capsule is printed for a patient, that safeguards the drug through the stomach and then releases it slowly over the length of the small intestine to provide a prolonged therapeutic effect—this could improve adherence and therapeutic outcomes for medications that currently require multiple doses per day.

### 4.1. Implications for Precision Medicine and Pharmaceutical Manufacturing

The successful demonstration of tunable release profiles from 3D-printed capsules underscores the potential of this technology in precision medicine. In a personalized healthcare scenario, pharmacists in a compounding pharmacy or hospital could adjust the capsule shell formulation (or geometry) on demand to craft a medication that matches an individual patient’s pharmacokinetic requirements [17]. For example, a patient who metabolizes a drug rapidly might benefit from a capsule that releases the drug more slowly to maintain adequate plasma levels, whereas another patient might need a quicker release due to a slow absorption rate. Our two prototype formulations illustrate how one could easily modify the release: a higher HPMC-AS content yields a slower release suitable for sustained therapy, whereas a higher PEG content speeds up release for cases needing quicker onset. The iterative optimization of capsule design (varying polymer ratios, shell thickness, infill density, etc.) can be guided by in vitro testing to achieve the desired profile before printing the final patient-specific batch. This level of control is a significant step towards truly individualized dosing regimens, moving away from the current paradigm where patients often adjust to the medicine rather than medicines being adjusted to the patient [40].

Beyond patient-specific therapy, there are practical manufacturing implications. The use of FFF 3D printing for capsules could streamline small-scale production and circumvent some limitations of capsule manufacturing. Traditional capsule making (for gelatin capsules) is a high-volume industrial process not easily adaptable to small-batch personalization. In contrast, 3D printing can produce small batches (even single units) economically and with minimal waste [13]. We utilized readily available pharmaceutical polymers and a benchtop printer to produce functional capsules, highlighting that the barrier to entry for this technology is relatively low. Moreover, because our capsules do not rely on a separate coating step (the functionality is built into the shell formulation), production is simplified—essentially a one-step process after filament preparation. Melt extrusion printing processes like this have been touted as a paradigm shift for on-demand manufacturing of modified-release products [18]. Regulatory science is beginning to catch up: the FDA has shown openness to 3D-printed drug products (as evidenced by Spritam’s approval and guidance documents on personalized therapies), though challenges remain in ensuring quality and consistency for printed medicines [19,41].

Our study also offers a proof-of-concept for polymer blend optimization in printed dosage forms. By demonstrating that polymer ratio adjustments can fine-tune release, we provide a blueprint for customizing other release profiles. For instance, if an even more prolonged release is needed, one could explore higher molecular weight polymers or additional release retardants in the shell. Conversely, for near-immediate release after a delay, one might incorporate pore formers that rapidly leach out at intestinal pH. The flexibility in excipient selection is vast, given that 3D printing can accommodate polymers not feasible in classic capsule making. We used HPMC-AS and PEG, but alternatives like Eudragit derivatives, enteric starches, or even novel pH-responsive hydrogels could be employed depending on the drug’s properties and target release site [42].

### 4.2. Potential Clinical and Industrial Impact

From a clinical perspective, sustained-release enteric capsules could improve therapy for conditions where maintaining drug levels is important while avoiding gastric irritation or degradation. For example, certain anti-inflammatory drugs cause stomach irritation; an enteric sustained capsule could release the drug slowly in the intestine, minimizing peak concentrations that cause irritation and eliminating drug exposure to the stomach altogether. Another scenario is treating chronic conditions (like arthritis or hypertension) with medications that currently require multiple daily doses: a personalized capsule could be printed to release the drug throughout the day after passing the stomach, effectively acting as a customized once-daily pill. Additionally, pediatric and geriatric patients who often require dose adjustments might benefit from these tailored capsules; the approach could reduce the need for pill splitting or multiple formulations by consolidating therapy into one bespoke capsule [43]. Indeed, the literature suggests that pediatric pharmacotherapy could be significantly advanced by 3D printing, as it allows for dosage forms that account for a child’s taste preferences, swallowing ability, and dosing needs [28]. While our work did not specifically address taste-masking, the principle of customizing release and composition could be extended to create child-friendly, sustained-release formulations.

On the manufacturing side, adopting 3D printing for pharmaceuticals requires overcoming challenges like ensuring dose uniformity, print fidelity, and regulatory validation of each printed lot [7]. Our results for weight uniformity and dissolution reproducibility are encouraging in this regard—they show that with proper formulation and calibration, printed capsules can meet standard quality criteria. The scale-up of such technology might involve parallel printing (multiple printer arrays) or faster printers (e.g., using larger nozzles or multiple nozzles working concurrently) to produce larger quantities when needed [21]. However, the true value of this approach may lie in decentralized manufacturing: pharmacies or hospitals could produce capsules on-site, thereby reducing the need for large-scale production and storage of multiple dosage strengths [44]. This aligns with the vision of “hospital pharmacy manufacturing” where 3D printers formulate medications tailored to each prescription [10,21].

### 4.3. Limitations and Future Research Directions

While this study provides valuable insights, it has certain limitations. First, the evaluation was conducted in vitro; thus, in vivo pharmacokinetic studies will be needed to confirm that the observed sustained release translates to prolonged drug absorption and action in humans or animal models. Factors such as gastric emptying time variability and intestinal motility could influence the real-world performance of these capsules. For instance, if a capsule transits very quickly, a formulation intended to release over 5–6 h might reach the colon (where pH and fluid volume are different) before fully releasing, potentially affecting bioavailability. Future work could involve in vivo–in vitro correlation (IVIVC) studies to fine-tune the release duration to physiological transit times.

Second, our polymer blends, while effective, are relatively simple. More complex formulations could be explored to further customize release behavior—for example, incorporating swellable erodible polymers (such as HPMC or xanthan gum) to achieve near-zero-order release, or using enteric polymers with higher pH thresholds to target release to the colon (pH~7.0–7.5) [5]. Additionally, the approach could be extended to multi-layer or multi-compartment capsules. Li et al. reported a 3D-printed compartmentalized capsular device with separate drug compartments that enabled a two-pulse release [36]. Such architectures, combined with our polymer blend strategy, could yield capsules that first release one dose (or drug) in the intestine and another dose later in the colon, for example. This is particularly interesting for multi-drug regimens or chronotherapy (e.g., a morning dose and an afternoon dose in one capsule).

Another direction is to investigate different drugs with optimized capsule shells. Acetaminophen was chosen for its well-characterized dissolution and ease of analysis, as it is highly soluble and fast-absorbing. Testing a poorly soluble drug or a drug with a narrow absorption window could reveal how the matrix release interacts with drug solubility constraints. If a drug has solubility issues, one might include solubilizers in the fill or use a self-emulsifying drug delivery system (SEDDS) inside the capsule, as was conducted by Shaqour et al. for ketoprofen [29]. Such combinations of formulation strategies (controlled-release shell + enhanced-release fill) could maximize oral bioavailability for challenging compounds.

From a technological standpoint, improvements in 3D printing hardware could enhance the quality and functionality of printed capsules. Higher-resolution printers or those capable of multi-material printing will enable thinner shells or gradient compositions where the interior of the shell has a different composition than the exterior (for example, a higher HPMC-AS concentration on the outer layer for strong enteric protection, and higher PEG inside for quicker core release). This type of graded structure is uniquely achievable by additive manufacturing and could further optimize the release kinetics, perhaps producing an initial slow release followed by faster release later, or vice versa, depending on therapeutic needs [21,45].

### 4.4. Future Outlook

The success of our optimized 3D-printed capsules in achieving sustained and targeted release paves the way for bespoke drug delivery systems that align with the principles of precision medicine. As digital health and personalized therapy advance, one can envision an ecosystem where a patient’s pharmacological profile, including genetics, metabolism, and disease state, is used to design a custom capsule via software, which is then printed at the point of care. Recent clinical studies have already trialed 3D-printed formulations in patients (e.g., Goyanes et al.’s study on tailored isoleucine tablets for metabolic disorder patients [12]), demonstrating both feasibility and positive outcomes. To move toward that reality for capsules, further interdisciplinary collaboration will be important—combining pharmaceutical sciences with materials engineering and data science.

Regulatory frameworks will need to adapt to such on-demand manufacturing. Each personalized capsule might be considered a batch of one, challenging traditional quality control methods. One solution could be to integrate real-time release testing and process analytical technology (PAT) into printers, ensuring that each printed capsule meets the specifications (e.g., via inline near-infrared spectroscopy to verify composition). Another aspect is patient acceptance: both healthcare providers and patients will need to trust these novel dosage forms. Education and evidence of safety/efficacy will play a role in adoption.

As 3D printing is poised to significantly augment the toolkit for developing advanced oral drug delivery systems, our work provides a concrete example of how formulation optimization in a 3D-printed capsule can yield a therapeutic advantage—combining the benefits of enteric protection and sustained release in a single, customizable platform. As the manufacturing technology continues to mature, one can anticipate a new generation of “smart” capsules that not only release drugs at the right place and pace but could even respond to physiological cues (with appropriate material choices) to further fine-tune therapy. The integration of such dosage forms into mainstream pharmaceutical practice could lead to more effective treatments with fewer side effects, ultimately aligning medication regimens more closely with individual patient needs.

## 5. Conclusions

This study demonstrated the successful optimization of 3D-printed capsule shells for sustained and targeted drug release, using polymer blends of HPMC-AS, PEG, and PVA to create enteric and controlled-release functionality in a single unit. The 3D-printed capsules showed zero release in acidic conditions and a gradual, extended release in the intestinal pH, in contrast to commercial enteric gelatin capsules, which rapidly released their drug load after the pH trigger. By comparing two formulations, we illustrated how tuning the polymer ratio modulates the release rate: a higher HPMC-AS content led to a more prolonged release, whereas higher PEG content accelerated the release, though still in a sustained manner. All formulations met quality benchmarks for weight uniformity and achieved complete or near-complete drug release within the test duration, indicating the reliability of the printed capsules.

The implications of these findings are significant for the advancement of personalized medicine. The ability to customize capsule release profiles means that therapy can be tailored to the individual patient’s needs—for instance, providing longer-lasting symptom control or targeting drug delivery to specific regions of the GI tract. Patients could receive bespoke medications that improve efficacy and safety, such as avoiding peak-related side effects or ensuring a drug is released only where it can be absorbed best. Importantly, the 3D printing approach allows such customization to be carried out on demand, potentially by a pharmacist, rather than requiring new manufacturing lines for each variant.

From a clinical standpoint, a 3D-printed sustained-release enteric capsule could benefit numerous scenarios: delivering acid-sensitive but slow-acting drugs (e.g., certain peptides or probiotics) past the stomach, maintaining steady drug levels for chronic disease management, or combining multiple dosing steps into one device. For example, a patient with inflammatory bowel disease might take a single printed capsule that first protects a drug through the stomach, then releases it gradually in the small intestine to reduce systemic peaks, and perhaps even a portion releases in the colon to target local inflammation—all in one capsule. Such sophistication is challenging to achieve with the current dosage forms, but it is made feasible by 3D printing technology.

The study also contributes to the growing evidence that 3D printing can produce high-quality pharmaceutical products. The printed capsules were robust, exhibited reproducible performance, and utilized materials already familiar in the pharmaceutics field. This convergence of novel manufacturing with known excipients may ease the regulatory pathway for future products. Indeed, regulatory agencies are actively exploring how to evaluate and approve 3D-printed drug products [19], and our work provides a case study showcasing quality-by-design in a printed formulation [3] (where polymer choice and ratio serve as critical quality attributes determining the release profile).

In conclusion, we demonstrated that 3D-printed capsule shells can be fine-tuned to achieve desired drug release patterns that surpass what is possible with standard capsule designs. The optimized capsules combine the roles of an enteric protector and a sustained-release matrix, embodying a multifunctional oral delivery system. This work lays a foundation for the future development of patient-centric therapies, where dose, release rate, and treatment scheduling can be personalized with a simple change in a digital design or formulation recipe. Further research and development, including in vivo studies and expansion to other drugs, will pave the way for translating this promising technology into real-world healthcare solutions. As 3D printing continues to penetrate the pharmaceutical industry, it holds the promise of fundamentally changing how medicines are designed, manufactured, and dispensed—ultimately heralding an era of truly individualized pharmacotherapy.

## Figures and Tables

**Table 1:** Compositions of extruded filaments using the hot melt extrusion (HME) process.

	Polymer	Plasticizer	Adhesive	HME Temp. (°C)	FFF Printing Nozzle Temp. (°C)
Blend A	80% HPMC-AS	15% PEG 4000	5% PVA	175	185
Blend B	70% HPMC-AS	20% PEG 4000	10% PVA	175	185

**Table 2:** Optimized 3D printing parameters for pharmaceutical capsule fabrication.

Parameter	Blend A (80:15:5, HPMC-AS:PEG)	Blend B (70:20:10, HPMC-AS:PEG)
Nozzle temperature (°C)	210	205
Build plate temperature (°C)	60	60
Print speed (mm/s)	20	20
Layer height (mm)	0.1	0.1
Infill density (%)	100 (solid walls)	100 (solid walls)
Actual printed capsules	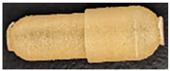	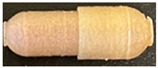

Note: Blend B used a slightly lower nozzle temperature due to the higher PEG content.

**Table 3:** Goodness-of-fit (R^2^) values for kinetic models fitted to in vitro dissolution profiles.

Model	Blend A	Blend B	Commercial Capsule
Zero-order (R^2^)	0.892	0.830	0.286
First-order (R^2^)	0.621	0.546	0.855
Second-order (R^2^)	0.344	0.279	0.963
Higuchi (R^2^)	0.959	0.918	0.566
Korsmeyer–Peppas (R^2^)	0.995	0.991	0.860

## Data Availability

The original contributions presented in this study are included in the article. Further inquiries can be directed to the corresponding authors.

## Abbreviations

The following abbreviations are used in this manuscript:

3D Printing	Three-Dimensional Printing
FFF	Fused Filament Fabrication
HPMC-AS	Hydroxypropyl Methylcellulose Acetate Succinate
PEG	Poly(ethylene glycol)
PVA	Poly(vinyl alcohol)
SGF	Simulated Gastric Fluid
SIF	Simulated Intestinal Fluid
UV	Ultraviolet
PBS	Phosphate Buffer Saline
API	Active Pharmaceutical Ingredient
DE	Dissolution Efficiency
ANOVA	Analysis of Variance
FDA	Food and Drug Administration
EMA	European Medicines Agency
IVIVC	In Vivo–In Vitro Correlation
USP	United States Pharmacopeia
RSD	Relative Standard Deviation
TGA	Thermogravimetric Analysis
PAT	Process Analytical Technology
NSAID	Non-Steroidal Anti-Inflammatory Drug
